# RibDif: can individual species be differentiated by 16S sequencing?

**DOI:** 10.1093/bioadv/vbab020

**Published:** 2021-09-19

**Authors:** Mikael Lenz Strube

**Affiliations:** Department of Biotechnology and Biomedicine, Technical University of Denmark, Kongens Lyngby DK 2800, Denmark

## Abstract

**Motivation:**

Metataxonomic analysis is now routinely used to profile the microbiome of virtually every ecological niche on planet Earth. The use of amplicon sequence variants (ASVs), proposing to be the exact biological 16S rRNA amplicon sequences of a given biological system, is now considered the gold standard. However, the 16S rRNA genes, and in particular the amplicons derived from it, are not unique for most species nor are they necessarily unique within individual genomes. Despite these restrictions, individual ASVs are often used to make inferences on the state of a given ecosystem, which may cause erroneous conclusions on the effects of a given species on a specific host phenotype or ecosystem.

**Results:** To support researchers working with metataxonomics, we have developed RibDif, which easily and rapidly evaluates the feasibility of using metataxonomics to profile individual species. We use RibDif to demonstrate that the genus Pseudoalteromonas contains species that are impossible to distinguish with 16S amplicons and that this is a common motif in bacterial genera. We propose that researchers consult RibDif when making conclusions on individual species from metataxonomic data.

**Availability and implementation:**

RibDif is freely available along with source code and detailed documentation at https://github.com/mikaells/RibDif.

## 1 Introduction

The analysis of microbiomes has been vastly improved by high-throughput sequencing technologies and concurrent improvements in computational biology. Lately, the adaption of the amplicon sequence variant (ASV) rather than the operational taxonomic unit (OTU) theoretically allows researchers to resolve bacteria at the species level. Since the OTU is an artificially constructed consensus sequence (often 97% similarity), much information is lost compared to the ASV, which instead aims to represent the original biological sequence. The advantage here is evident, e.g. individual species or strains of a genus may have unique properties and ASVs are easier to follow across studies than OTUs (Callahan *et al.*, 2017). This, however, requires that a single sequence, even devoid of technical error, is unique for a particular species.

Unfortunately, many bacterial species have multiple different alleles (Větrovský and Baldrian, 2013) and the alleles within a genus may not be unique enough to distinguish species (Johnson *et al.*, 2019). The first issue may lead to multiple ASVs from single genomes and the second will not allow one to distinguish species at all. It has become commonplace to associate individual ASVs with ecosystem or host phenotype, but for the reasons given above, this may not be possible. Moreover, inferring the genome, and hence the metabolic capacity, from amplicons has had limited success (Sun *et al.*, 2020), assumedly for the same reasons. Lastly, diversity estimates become increasingly meaningless if some genomes have multiple alleles or if some species share the same unique allele.

To investigate this, we have developed RibDif, which allows users to easily and quickly answer if species within a given genus can be resolved using standard metataxonomic methods, hence letting users evaluate if ASVs are useful for their particular research question or if alternative methods are to be employed. As described previously, one solution is to develop primers not targeting the 16S region (Lauritsen *et al.*, 2021; Strube *et al.*, 2018).

The software provides a detailed analysis of individual genomes and graphical representations of overall 16S amplicon diversity as well as a report on exactly which species overlap.

## 2 Methods and implementation

The code for analysis is available at https://github.com/mikaells/RibDif. Given a genus or a species, the software downloads all available complete genomes from NCBI using ncbi-genome-download (https://github.com/kblin/ncbi-genome-download). All 16S rRNA genes at least 90% of full length are then found by Barrnap (https://github.com/tseemann/barrnap) and these sequences are next subjected to *in silico* polymerase chain reaction (PCR) using in_silico_pcr.pl (https://github.com/egonozer/in_silico_pcr), by default using primers targeting the V3V4-region (CCTACGGGNGGCNGCAG and GACTACNNGGGTATCTAATCC) and full-length V1V9-region (AGRGTTYGATYMTGGCTCAG and RGYTACCTTGTTACGACTT), allowing one mismatch and one insertion. User-defined primers are allowed using the –p/–primer argument. Optionally, the resulting amplicons of all genomes are aligned by use of MAFFT (Katoh *et al.*, 2002) using the —adjustdirection option and a corresponding tree is generated with fasttree (Price *et al.*, 2010). Optional detailed intragenomic analysis is performed by pyani (https://github.com/widdowquinn/pyani).

Next, VSEARCH (Rognes *et al.*, 2016) is used to cluster the amplicons at 100% identity (as is the theoretical cutoff for ASVs). These clusters are then tabulated across genomes, after which a summary and graphics of allele multiplicity and textual information of species overlap are provided.

The software is implemented in bash and R and is fully parallelized using GNU parallel (Tange, 2020). RibDif with default settings will analyze the *Escherichia* genus, consisting of 1700 genomes, in 1481 s, of which genome download (108 s) and rRNA prediction (860 s) are the major bottlenecks on a 10× dual-core Intel(R) Xeon(R) W-2155 CPU @ 3.30 GHz. Analysis of the Pseudoalteromonas genus, consisting of 58 genomes, is accomplished in 62 s.

## 3 Results

The analysis relies on the RibDif software described in Section 2. Briefly, the software downloads complete genomes of a genus/species, extracts the 16S rRNA genes, amplifies them *in silico* and clusters the amplicons at 100% identity to mimic the concept of an ASV.

Three problematic scenarios are observed. A genome may have 16S rRNA amplicons (i) that are the same in different species, (ii) have multiple variants and (iii) may have multiple variants that are individually shared with other species.

**Fig. 1.**
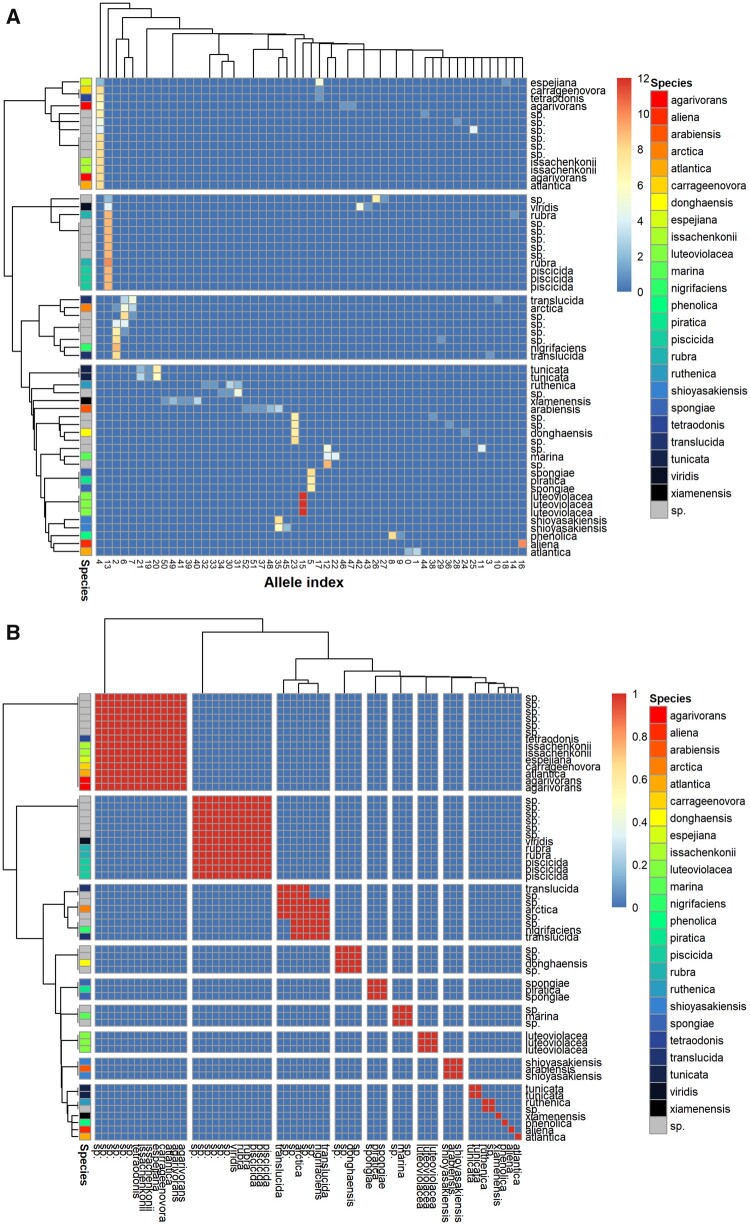
Heatmaps of 16S rRNA V3V4 alleles in *Pseudoalteromonas* as generated by RibDif. V3V4 amplicons were generated from the 16S rRNA gene of *Pseudoalteromonas* and clustered at 100% identity. (**A**) Alleles are across columns and individual genomes on the rows. The count of members in a given cluster is given as color intensity. Rows and columns are additionally clustered by similarity in cluster abundance using Wards method. (**B)** Confusion matrix of each genome showing which will overlap in V3V4-based metataxonomy, clustered using Wards method. The figure is generated using ‘RibDif –g Pseudoalteromonas’. sp., genomes only classified as *Pseudoalteromonas* species.

### 3.1 Pseudoalteromonas V3V4 amplicons

As an example, consider the genus *Pseudoalteromonas*, a marine bacterium with 48 recognized taxa of which 24 have published complete whole genomes (36 in total). In addition, 22 complete genomes of unspecified species are available. When clustering the 499 V3V4 amplicons derived from these genomes at 100% identity into 53 unique alleles, it is observed that several of these are identical across species (Fig. 1A). Specifically, *Pseudoalteromonasrubra*, *Pseudoalteromonasviridis* and *Pseudoalteromonaspiscicida* are indistinguishable as they share allele 13. In addition, a genome of *P.viridis* contains three unique alleles, one of which is also found in allele 13. Allele 4 is represented in genomes of *Pseudoalteromonasespejiana, Pseudoalteromonasissachenkonii*, *Pseudoalteromonasatlantica*, *Pseudoalteromonascarragenenovora*, *Pseudoalteromonasagarivorans* and *Pseudoalteromonastetradonis*, several of which contain multiple other alleles. As seen in Figure 1A and B, a genome of *Pseudoalteromonasarctica* (along with several unclassified species) contains three alleles, each of which overlap separately with genomes of *Pseudoalteromonastranslucida* and *Pseudoalteromonasnigrifaciens*. Genomes such as the one representing *P.espejiana*, which have two shared but one unique allele, are still problematic as all alleles are amplified by PCR and the shared alleles (alleles 4 and 17) will incorrectly suggest the presence of 5 and 2 additional species, respectively.

### 3.2 Full-length V1V9 amplicons

**Fig. 2.**
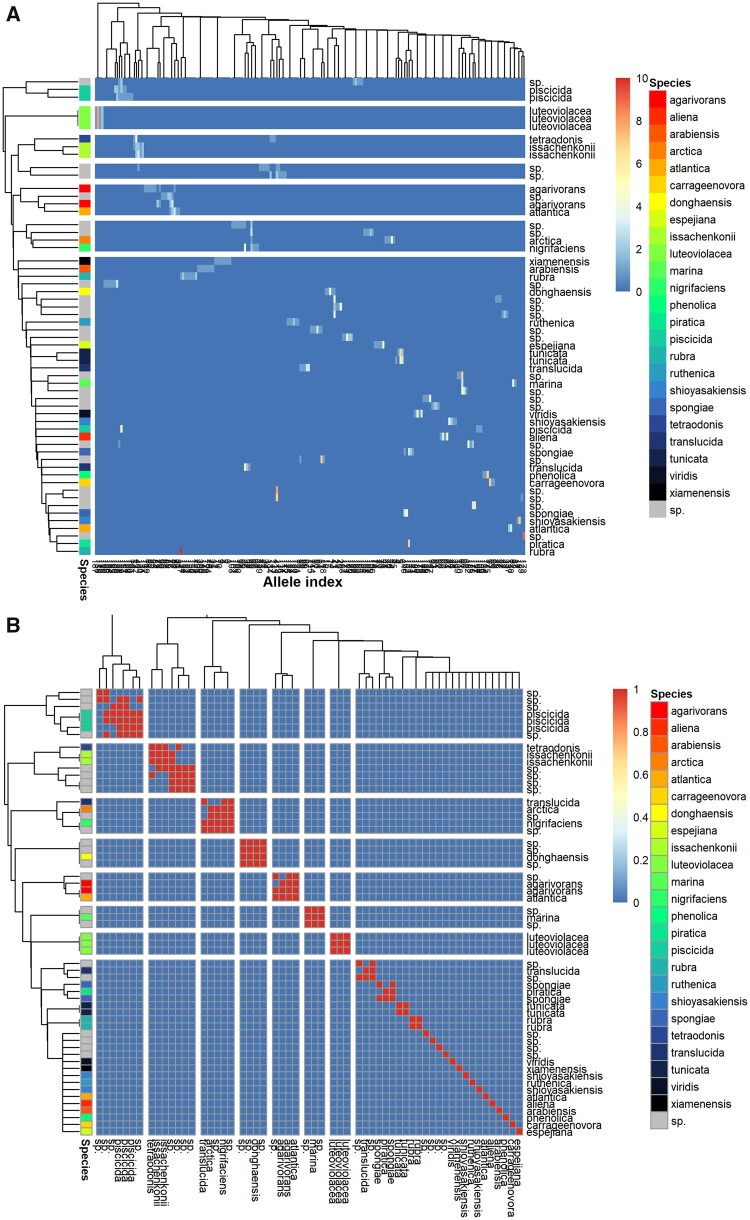
Heatmaps of 16S rRNA V1V9 alleles in *Pseudoalteromonas* as generated by RibDif. V1V9 amplicons were generated from the 16S rRNA gene of *Pseudoalteromonas* and clustered at 100% identity. (**A**) Alleles are across columns and individual genomes on the rows. The count of members in a given cluster is given as color intensity. Rows and columns are additionally clustered by similarity in cluster abundance using Wards method. (**B**) Confusion matrix of each genome showing which will overlap in V1V9-based metataxonomy, clustered using Wards method. The figure is generated using ‘RibDif –g Pseudoalteromonas’. sp., genomes only classified as *Pseudoalteromonas* species.

When analyzing full-length V1V9 amplicons rather than the V3V4 amplicons, substantially more (202) unique alleles are generated owing to the sequences being approximately 3 times longer (Fig. 2). The resolving power of these amplicons is substantially improved, e.g. *P.rubra*, *P.viridis* and *P.piscicida* are now distinguishable and all alleles of *P.viridis* are unique. However, the following pairs of species are still indistinguishable: *tetraodonis*/*issachenkonii, translucida/arctica/nigifaciens*, *agarivorans/atlantica* and *spongia/piratica.*

### 3.3 Medically and biotechnologically important genera

Other genera, such as *Lactobacillus*, are easier to distinguish (Table 1). Of the 19 species having a completed whole genome, the only overlaps in V3V4-amplicons are in the known closely related species of *gasseri/paragasseri* and *amylovorus/ultunensis*, although the latter are distinguishable with V1V9-amplicons. On the other hand, other genera investigated show the same pattern as in *Pseudoalteromonas*, e.g. high allele multiplicity and extensive species overlap, which, however, is somewhat mitigated by using full-length V1V9 amplicons. The *Pseudomonas* genus is particularly difficult to separate into species, being both unusually large (92 species) as well as having 80.43% species overlap. It is likely that most genera are not possible to resolve into species level using V3V4 amplicons, although the use of V1V9-amplicons decreases overlap substantially in some genera, such as *Vibrio* and *Staphylococcus*.

**Table 1.** Medically and biotechnologically relevant genera and their resolvability using 16S amplicon sequencing of the V3V4 and V1V9 region

Genus	Complete genomes	Species	Allele multiplicity^a^ (%)		Overlaps^b^ (%)	
			**V3V4**	**V1V9**	**V3V4**	**V1V9**
*Lactobacillus*	123	19	30.08	62.60	21.05	10.53
*Vibrio*	336	52	86.61	98.51	57.69	17.31
*Bacillus*	856	47	63.90	93.93	74.47	55.32
*Pseudomonas*	860	92	24.53	59.42	80.43	35.87
*Staphylococcus*	889	38	34.76	87.49	65.79	13.16
*Escherichia*	1700	4	32.94	91.47	100.00	50.00

a The percentage of genomes having more than one unique allele.

b The percentage of species having at least one allele overlapping another species.

## 4 Discussion

The advantages of ASVs are evident (Callahan *et al.*, 2017). The use of original biological sequences of a sample rather than artificial consensus sequences theoretically allows one to describe the species of a microbiome with higher resolution. Unfortunately, it does not appear that all species can be described as such.

In this analysis, we have observed some issues with the ASV logic as previously described (Johnson *et al.*, 2019; Větrovský and Baldrian, 2013). It is observed that individual bacteria may have multiple 16S genes and, importantly, may have multiple amplicon variants within the V3V4 region. More problematically, several of these are shared between individual species in several major bacterial genera.

As seen from the analysis of *Pseudoalteromonas*, several practical issues can appear. A researcher analyzing a community containing *P.viridis* using V3V4 metaxonomics will—assuming complete absence of technical error—observe three ASVs, which will overinflate the *Pseudoalteromonas* richness and incorrectly suggest the presence of *P.rubra* and *P.piscicida*. In a natural system with potentially 1000 s of species across multiple genera, these issues will evidently disturb conclusions made on both diversity and species presence/absence. Condensing ASVs into species or genera may be a more robust option than relying on ASVs as individually meaningful units.

Researchers should consider these issues when evaluation metataxonomic data and we propose they consult RibDif before making conclusions on individual ASVs. Researchers being particularly interested in species differentiation should consider full-length V1V9 amplicons or primers tailored to individual genera as previously done for *Pseudomonas* (Lauritsen *et al.*, 2021) and *Staphylococcus* (Strube *et al.*, 2018).
